# Cortical Excitability across the ALS Clinical Motor Phenotypes

**DOI:** 10.3390/brainsci11060715

**Published:** 2021-05-28

**Authors:** Thanuja Dharmadasa

**Affiliations:** 1 Nuffield Department of Clinical Neurosciences, Oxford University, Oxford OX3 9DU, UK; thanuja.dharmadasa@ndcn.ox.ac.uk; 2 Brain and Mind Centre, Sydney Medical School, University of Sydney, Sydney, NSW 2050, Australia

**Keywords:** amyotrophic lateral sclerosis, phenotypic heterogeneity, cortical hyperexcitability, transcranial magnetic stimulation, ALS focality, survival

## Abstract

Amyotrophic lateral sclerosis (ALS) is characterized by its marked clinical heterogeneity. Although the coexistence of upper and lower motor neuron signs is a common clinical feature for most patients, there is a wide range of atypical motor presentations and clinical trajectories, implying a heterogeneity of underlying pathogenic mechanisms. Corticomotoneuronal dysfunction is increasingly postulated as the harbinger of clinical disease, and neurophysiological exploration of the motor cortex in vivo using transcranial magnetic stimulation (TMS) has suggested that motor cortical hyperexcitability may be a critical pathogenic factor linked to clinical features and survival. Region-specific selective vulnerability at the level of the motor cortex may drive the observed differences of clinical presentation across the ALS motor phenotypes, and thus, further understanding of phenotypic variability in relation to cortical dysfunction may serve as an important guide to underlying disease mechanisms. This review article analyses the cortical excitability profiles across the clinical motor phenotypes, as assessed using TMS, and explores this relationship to clinical patterns and survival. This understanding will remain essential to unravelling central disease pathophysiology and for the development of specific treatment targets across the ALS clinical motor phenotypes.

## 1. Introduction

**Figure 1:**
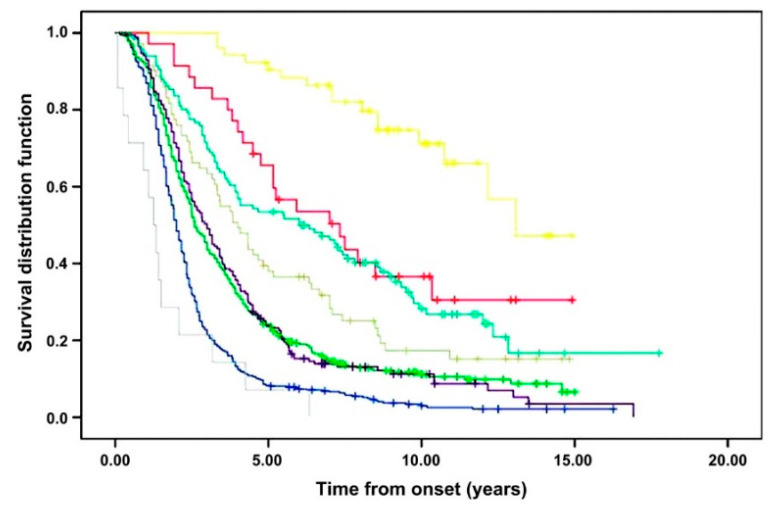
Survival according to ALS clinical motor phenotype (in an Italian cohort). PLS, yellow; PMA, red; UMN-predominant ALS, turquoise; flail arm, light green; flail leg, mint green; classical ALS, purple; bulbar-onset (classical ALS), blue; respiratory-onset (classical ALS), grey. Reprinted with permission from Chio et al. [4]. Copyright 2011 BMJ Publishing Group Ltd.

Although the clinical manifestations of amyotrophic lateral sclerosis (ALS) largely result from progressive degeneration of the human motor system, there is marked phenotypic heterogeneity between cases and a wide range of unexplained clinical severity [1,2,3]. While phenotypic complexity is increased by the presence of non-motor symptoms such as frontotemporal dementia, the selectivity for the motor system remains the clinical hallmark of the disease. The clinical motor phenotypes reflect the in vivo anatomy of underlying neuropathology; their differences are attributable to motor degeneration occurring with strikingly dissimilar onset, severity, and rate across upper motor neuron (UMN) and lower motor neuron (LMN) levels [1]. Population-based studies have identified that these clinical subgroups have important diagnostic and prognostic significance, highlighting differences in their patterns of disease spread and clinical outcomes (Figure 1) [2,3,4]. Such clinical distinctions have questioned underlying disease biology and whether motor phenotypes represent multiple diseases with different mechanisms, or rather, exist within a spectrum of one disease that shares a final common pathogenic pathway. Threads of continuity between the clinical syndrome, the neuropathological lesion, and genetics continue to provide support toward the latter within the infrastructure of a multistep, multisystem etiological model of disease, but such questions still remain unsettled.

Many of the earliest clinical observations in ALS reflect features that seem to subserve the dysfunction of an expanded cortical motor system in humans [5,6,7]. The onset of motor weakness is typically focal, but the literature on focality has been largely descriptive of LMN features alone [1,5,8]. This is partly due to the inherent difficulties of detecting clinical UMN signs, which can be subtle during early disease stages and obscured by severe muscle wasting in later stages, and partly due to the lack of a universally accepted cortical biomarker [8,9]. As such, the cortical changes underlying clinical weakness have remained challenging to identify in vivo, but they are increasingly postulated as a critical determinator of disease onset and patterns of spread [5,7,10]. Progress in the ability to quantify the UMN lesion is now providing more opportunities to resolve this hypothesis [9,11]. In particular, neurophysiological exploration of the motor cortex with transcranial magnetic stimulation (TMS) has suggested the early presence of cortical hyperexcitability, which appears linked to clinical site of onset [12,13,14]. This technique measures axonal and synaptic excitability through non-invasive electromagnetic interrogation of the corticomotoneuronal system [15], and such findings in ALS patients are likely to be a result of several mechanisms that affect the critical balance between cortical inhibition and facilitation in the primary motor cortex [12,16,17]. In turn, this may contribute to glutamate-mediated excitotoxicity, which is a potentially critical step in the pathogenic process [12].

Evidence of regional differences in cortical excitability across the ALS motor cortex have recently questioned whether cortical abnormalities are equally relevant to all clinical motor phenotypes and whether this is an important driver of the variable clinical outcomes [13,18,19]. This review will analyse the cortical excitability profiles across typical and atypical ALS clinical motor phenotypes, as assessed using TMS, and explore the relationship of this to the observed clinical patterns and survival. The nature of selective motor vulnerability and implications to disease pathophysiology will also be discussed.

## 2. Archetypical Clinical Motor Phenotypes

**Figure 2:**
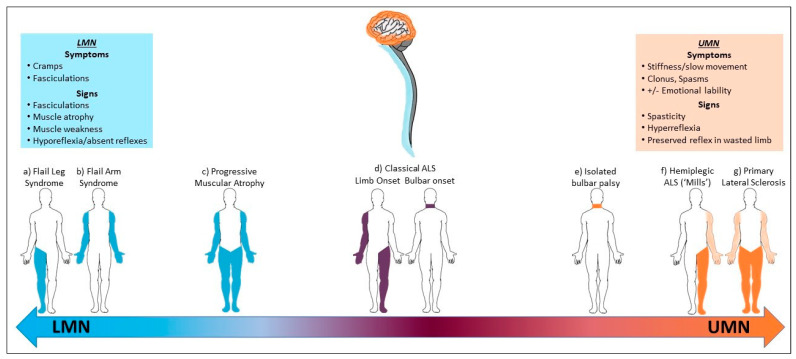
Pattern of motor involvement across the ALS clinical phenotypes. Blue, LMN involvement; Orange, UMN involvement; Purple, mixed (UMN/LMN) involvement.

The lexicon of phenotypic classifications has emerged through the evaluation of dominant clinical features, with distinctions made based upon two main anatomical factors: (i) the mix of UMN/LMN involvement and (ii) the initial area of clinical onset. As such, the clinical motor presentations of ALS exist on a continuum, with the spectrum extending from LMN only to UMN only signs (Figure 2, Supplementary Table S1) [2,4,20]. For the vast majority of all presentations (70–75%), weakness begins asymmetrically and focally, and it follows the ‘classical’ pattern:

- The definitive clinical characteristics of this typical ALS phenotype are the presence of a relatively equal burden of UMN and LMN signs coexisting within the same symptomatic area (Figure 2d) [4,21]. Clinical disease can initially manifest in one of four main regions (upper-limb, lower-limb, bulbar, respiratory/truncal), but weakness usually begins in a limb, while respiratory onset is rare (1–5%) [3,4]. Bulbar-onset occurs in 20% of this group and increases in frequency with increasing age, which may explain the reported predominance in females [22]. These patients have a worse prognosis than their limb-onset counterparts [4,23]. Median survival is approximately 3 years [21], while UMN- or LMN-predominant variants of this form usually have a slower rate of progression [4].

Atypical forms of the disease occur at either extreme of the clinical spectrum:

- Patients with a pure UMN syndrome that has progressed for at least 4 years in the absence of LMN signs are diagnostically termed primary lateral sclerosis (PLS) (Figure 2g) [24]. PLS uniquely represents a selective loss of precentral pyramidal (upper) motor neurons [24,25], and in some cases, this is sharply delineated by ‘knife edge’ focal atrophy on structural MRI and a ‘stripe’ of fluorodeoxyglucose hypometabolism in the precentral gyrus on PET studies [26,27]. Whether this is a separate disorder or a *forme fruste* of classical ALS continues to be debated [24,28,29]. PLS is rare, representing 2–5% of all cases [3,4,29,30]. These patients are consistently younger and have a predilection for symmetrical lower-limb disease onset, although a very rare asymmetrical subtype of progressive hemiplegia has been described by Mills (1% of cases) (Figure 2f) [31]. Spinobulbar spasticity emerges insidiously as a rule, and a slow rate of progression gives this the most favorable prognosis of all the clinical motor phenotypes, with some reports of normal life expectancy [4,30].
- Patients with a clinically pure LMN phenotype represent 5% of all cases, and this was first reported as ‘progressive muscular atrophy’ (PMA) by Aran in 1850 (Figure 2c) [32]. This phenotype has a higher occurrence in males [4,20]. When occurring in a more generalised form (i.e., when more than 50% of limb regions are affected), it follows a similar prognostic course to classic ALS, and approximately 30% of patients develop UMN symptoms within 18 months [33].

Finally, some patients develop a ‘restricted’ atypical variant in which symptoms remain regionally confined for a prolonged period:

- In patients with LMN-only symptoms (i.e., ‘PMA’), a ‘flail limb’ subgroup develops a clinical syndrome that remains restricted to either the upper limbs (flail arm syndrome, 5–6% cases; Figure 2b) or less commonly, to the lower limbs (flail leg syndrome, 3–5% of cases; Figure 2a) for at least 12 months [34]. The flail arm phenotype was first described as the ‘scapulohumeral variant of progressive muscular atrophy’, and it is represented by proximal wasting and weakness in the upper limbs, which typically evolves to involve both limbs symmetrically [34,35]. The flail leg syndrome, recognised by Pierre Marie and first described by his student Patrikios (the ‘Marie–Patrikios’ form), describes a distal onset of weakness that usually starts asymmetrically in the lower limbs. The flail limb subgroups are more common in men, particularly the flail arm phenotype (4:1) [34]. The natural history of these syndromes is better than for classical ALS: time to spread to a second region is longer (at least 18 months, with 27% of cases still confined to the onset limb(s) after 36 months), and overall prognosis is more favorable, with longer median survivals (Supplementary Table S1) [34,35].
- Isolated bulbar palsy (IBP) occurs in 1–4% of cases (Figure 2e) [36]. By definition, this differs from bulbar-onset ALS due to the restriction of progressive deficits to the bulbar area for 6 months or more, while limb strength remains preserved. Contention exists over whether this warrants nosological separation, as most eventually progress to the classical form of the disease [37], but the clinical pattern appears different [36]. Patients are commonly older and female. UMN bulbar symptoms predominate (e.g., spastic dysarthria, emotional lability), and survival in this group may be improved by at least 12 months compared to bulbar-onset ALS [36].

## 3. Cortical Excitability across ALS Motor Phenotypes

Single and paired-pulse TMS techniques exploring the ALS motor cortex have consistently identified an imbalance in cortical excitability compared to healthy controls, promoting dysfunction at both corticomotoneuronal and interneuronal levels [16,17,38,39]. In particular, a reduction of short interval intracortical inhibition (SICI), which is a marker of GABA_A_ergic inhibitory interneuronal function, accompanied by an increase in intracortical facilitation (ICF) in some studies, likely reflecting excitatory motor cortical circuits, are the main paired-pulse TMS biomarkers that have supported the early presence of relative motor cortical hyperexcitability in ALS [12,17,40,41,42]. Complementing these TMS findings, histopathological studies have identified a loss of parvalbumin-positive inhibitory interneurons from postmortem analysis [43], while neurobiochemical evidence has revealed a reduction in GABA levels and an increase in glutamate and glutamine levels in the ALS motor cortex [44]. Single-pulse TMS paradigms measuring resting motor thresholds (RMT) and the cortical silent period (CSP) have also demonstrated intrinsic cortical abnormalities to supplement this concept, with variable changes reported in these parameters depending on clinical features and disease stage [45]. CSP has both spinal and cortical contributions but is predominantly determined by cortical inhibitory circuits acting via GABA_B_ receptors, distinct to those mediating SICI. This may be shortened in early stages of the clinical disease, which is likely to represent dysfunction and/or degeneration of GABAergic neurotransmission [39,46]. RMT is influenced by excitatory interneuronal circuits and dynamically evolves with disease progression, reflecting the excitability of corticomotoneurons to TMS [18,39,45,47]. Finally, the prolongation of central motor conduction time (CMCT) has additionally indicated dysfunction of the pyramidal tracts in ALS, in keeping with recognised extensive degeneration of myelinated fibers in lateral corticospinal tracts on histopathological review [48].

The UMN pattern of involvement in ALS seems to be an important variable controlling patterns of disease onset and spread, and it appears to underlie the development of specific clinical features such as the split hand [39]. The degree of hyperexcitability is also related to prognosis, with reduced intracortical inhibition identified as an independent survival factor [18,19]. Regional differences in cortical excitability across the motor cortex may signify different patterns of selective vulnerability across the phenotypes, which may mediate the observed variability of clinical disease evolution. The cortical excitability profiles (as seen using TMS) associated with the clinical motor phenotypes and the relationship to their disease trajectories will be discussed below (Table 1) [16,39].

### 3.1. Classical ALS Phenotypes and ‘Cortical Focality’

The classic features of cortical hyperexcitability have been observed using TMS in the typical ALS phenotype regardless of the initial site of onset [49]. Importantly, this central abnormality occurs early and has been linked to clinical onset, with UMN dysfunction observed prior to detectable LMN dysfunction in sporadic cases [50], before clinical onset in familial forms [49], and most prominently from regions corresponding to the side of disease onset [13,14]. These findings have promoted the concept of ‘cortical focality’, suggesting a discrete region of hyperexcitability within the motor cortex from which the disease may initially manifest [5,8]. This is supported by emerging neurophysiological evidence demonstrating regional cortical abnormalities that mirror the focality of symptom onset in classical ALS limb-onset groups, particularly when captured within the first 12 months of clinical disease [13,51]. With disease spread, cortical dysfunction continues to predominantly correspond to clinically affected regions, supporting the importance of corticomotoneuronal dysfunction in mediating the subsequent spread of clinical symptoms [13,14,39]. This demonstrable relationship between cortical dysfunction and clinical variables via TMS has been further buttressed by findings on structural MRI, which show focal cortical atrophy in areas of the motor homunculus corresponding to the clinical area of onset as well as strikingly linear associations between grey matter volume and clinical measures [52,53].

The degree of hyperexcitability appears to differ between bulbar-onset and limb-onset subgroups in classical ALS, with greater reduction in averaged SICI reported in the former [13,18]. This is similar to findings from magnetic resonance spectroscopy (MRS) showing a greater loss of neuronal integrity within the primary motor cortex, as measured by *N*-acetylaspartate (NAA) levels, as well as more extensive atrophy and white matter change in extra-motor and subcortical regions in the bulbar-onset subgroup than is seen for their limb-onset counterparts [54,55]. This increased cortical hyperexcitability linked to a generally increased burden of cortical involvement in the bulbar subgroup may offer a cortical explanation for their more malignant prognosis [19].

### 3.2. Atypical Phenotypes

The most unifying cortical abnormality across all atypical clinical phenotypes is decreased cortical inhibition, as captured by paired-pulse TMS analysis of SICI and ICF, signifying interneuron dysfunction akin to classical ALS [36,56,57,58,59,60]. This is supported by histopathological analysis [43,61], and PET studies in PLS [62]. It is interesting to note that the reduction in SICI may be less profound for atypical phenotypes compared to classical ALS. This has been suggested in PLS [60] and demonstrated for flail limb phenotypes [57], while in IBP and flail-leg variants, these parameters are normal, while symptoms remain restricted or UMN signs absent [36,57]. The limited analysis of atypical variants and small group numbers has meant that these findings largely remain observational only, and future studies will be necessary to probe whether a potentially more favorable cortical profile may explain their typically better survival.

Single-pulse TMS analysis reveals further cortical abnormalities commensurate to that seen in classical forms of the disease. CMCT delay is found across all subgroups, including at least 50–60% flail limb phenotypes irrespective of the presence of UMN signs, implying dysfunction of pyramidal tracts regardless of clinical motor disease pattern [11,56,57,63]. In PLS, this parameter may be more prolonged than in classical ALS, which is in line with more delayed primary peaks on peristimulus time histograms [58,59]. Motor thresholds are also typically higher and inexcitability to cortical stimulation is more frequent in PLS (71% [60] vs. 10% in classical ALS [18]). Although PLS is underscored by classic neocortical ALS pathological changes (alongside relative preservation of lower motor neurons) [64], these TMS differences reflect the greater loss of corticomotoneuronal connections and more predominant UMN involvement in this phenotype. Overall, the similarities of cortical abnormalities reported across the ALS motor phenotypes support shared fundamental mechanisms, but the differing regions of neuronal burden in line with clinical patterns suggests some heterogeneity of underlying pathological processes.

## 4. Implications

The heterogeneity of the clinical motor phenotypes has presented significant challenges to the diagnosis, management, and prognostication of ALS patients. The ability to measure the anatomical motor pathways in vivo can now begin to clarify central mechanisms that were historically imputed from clinical findings alone. TMS studies unveil UMN involvement in atypical phenotypes that show little or no clinical evidence [17], and they suggest that the dysfunction of inhibitory GABAergic interneuronal circuits is a ubiquitous finding of the ALS motor cortex across all clinical motor presentations, which is critical to pathogenesis [12,65]. The neurobiochemical milieu of abnormal GABAergic neurotransmission is supported through PET studies using [^11^C]flumazenil (a benzodiazepine GABA_A_ receptor ligand) [66], MRS analysis [26,67], and histopathology [68], further promoting glutamate-mediated excitotoxicity as a potentially key mechanism across the clinical spectrum of this disease [68]. Taken together, the similarities of cortical dysfunction across the clinical phenotypes suggest that these groups exist within one ALS disease continuum. This unified framework is further complemented by the distinctive molecular signature found for almost all patients regardless of clinical presentation, in which cell nuclei forfeit their normal expression of TDP-43 [61]. Conversely, differences between clinical phenotypes in regard to regional excitability patterns support a selectivity of motor degeneration and demonstrate some underlying pathophysiological variability. The specific mechanisms of this remain to be clarified, but this insight may eventually underpin clinical decision making and stratification for clinical trials. Studies of atypical phenotypes are still limited and groups sizes remain small, requiring further longitudinal analysis to establish this prospectively. The following will discuss the clinical and prognostic implications of cortical excitability patterns across the motor phenotypes.

### 4.1. Clinical Implications

Ravits and La Spada proposed that ALS starts focally in the motor nervous system based on the somatotopic anatomy of upper and lower motor neurons [1]. Although initial disease triggers remain to be fully elucidated, neurophysiological exploration across the motor cortices reinforce this careful clinical observation [5,8]. Focal abnormalities of motor cortical excitability topographically relate to the site of limb onset in classical ALS, promoting a discrete cortical region that mirrors focal clinical change. This focal change may not be random, determined by age and gender, and influenced by genetics and environmental triggers [22]. Across clinical phenotypes, the selective involvement of distinct motor cortical regions relates to clinical symptomatology [3,13,51]. This offers hyperexcitability of specific corticomotoneuronal pathways as a critical mediator of variability in symptom development and clinical patterns, the pathophysiological basis of which may be linked to inhibitory postsynaptic potential mediated through GABA_A_ receptors [41]. The regional cortical differences of clinical phenotypes are also reported in combined neuroimaging [26] and neurochemical analyses of the neocortex [62,66], and they cumulatively highlight the importance of central dysfunction to the development of specific clinical disease patterns [1,5,8].

Central and peripheral propagation may occur in parallel or as a summated process, but ultimately, clinical disease progression correlates with the degree of cortical hyperexcitability and the level(s) of neuronal involvement [3]. Regarding the latter, when pathological burden is predominantly restricted to one motor pathway, such as in the flail limb phenotype (LMN pathway) or for PLS (UMN pathway), the rate of progression is significantly slower than classical (mixed pathway) ALS [3]. The distribution of cytopathology and neuronal loss in the cortex also follows this observation [61]. For example, flumazenil-PET studies show loss of GABAergic inhibitor receptor binding across motor regions in classical ALS, but there is relative preservation in slowly progressive atypical forms restricted to one motor pathway [66]. In such cases, the need and value of interventions, such as non-invasive ventilation or gastrostomy, is also not as clear as it is for classical ALS [2,69].

The attempt to illustrate corticomotoneuronal involvement in ALS across clinical motor phenotypes also raises several questions to be addressed. What is the biological interplay between UMN and LMN dysfunction in disease, and is it similar for all motor phenotypes? Why does the distribution of disease burden differ for atypical phenotypes, and how does their neuroanatomic propagation ensue across the motor levels? Why are some motor neurons specifically vulnerable to insults regionally, and why do some motor nuclei (such as sphincter and oculomotor) remain resistant or only occur relatively later (such as phrenic involvement)? As described in this review, the evidence of UMN involvement across all phenotypes, even in LMN-predominant forms that appear to lack clinical UMN signs, suggests that differences between clinical motor phenotypes may be based more on the anatomical distribution of pathological burden than on biological or molecular differences selecting one motor level over another [70]. As a single corticomotoneuronal input innervates several different spinal (alpha) motoneurons, it is possible that the dysfunction of relatively few cortical neurons may be enough to influence vulnerable spinal motor neurons in LMN predominant phenotypes [61]. Multicentric sites of onset have also been reasonably proposed, and they may link underlying region-specific cellular susceptibilities in upper and lower motor neurons. A LMN-driven process is harder to justify in these cases when contrasted with patients who have pure LMN-only diseases who do not show upstream evidence of corticomotoneuronal dysfunction [71]. Studies have additionally highlighted distinct morphological and functional characteristics of vulnerable motoneurons compared to more resistant motoneurons, implicating specific properties that may confer a unique sensitivity to excitability that may influence vulnerability to neurodegeneration [70]. Such factors modulating excitability in disease-vulnerable spinal motor neurons include their typically larger size, fast-fatigable subtype, and direct monosynaptic connection with corticofugal projection motoneurons, while resistant motoneurons are smaller, of the slow or intermediate (fast fatigue-resistant) subtype, and lack monosynaptic connections. Overall, such differences are likely to be governed within the complexities of the corticomotoneuronal–spinal cellular network, and improving this insight across motor phenotypes would provide a unique angle to understand the variable selectivity of motor pathways. Much remains to be untangled in future studies using careful prospective and objective interrogation across the motor axes. Identifying these differences is particularly relevant for ALS clinical trials, as inclusion of atypical motor trajectories has potentially obscured the effectiveness of therapy and increased the variability of response [72]. 

### 4.2. Prognostic Implications

Cortical excitability patterns may offer an important opportunity to help predict prognosis across the ALS phenotypes with greater accuracy, particularly when combined with other known clinical predictors of survival (such as older age at onset and respiratory dysfunction). The prognostic utility of averaged SICI has been demonstrated through correlation with measures of clinical disability and survival [12,13,14,19]. Greater cortical hyperexcitability is associated with a shorter survival, suggesting that decreased GABA at the level of the motor cortex is linked to a worse outcome [19]. Across classical ALS site-of-onset phenotypes, bulbar-onset patients appear to have the greatest reduction in SICI levels. This aligns with their more dramatic structural [73] and metabolic [54] abnormalities of the neocortex compared to limb-onset patients [26,67], suggesting a mechanistic link between cortical excitability, structural tissue integrity, and the development of clinical features [54]. Therefore, their more malignant prognosis, which is not completely explained by functional deficits, may be due to greater levels of transsynaptic glutamatergic excitotoxicity, driving the degeneration of motor neurons [5]. In atypical phenotypes, patients with evidence of hyperexcitability in flail leg phenotypes demonstrate a worse survival [57], while the suggestion of a less hyperexcitable motor cortex across the other subgroups may be relevant in view of their slower disease trajectories [57,60]. Ultimately, excitability profiles (particularly as measured by paired-pulse TMS) may offer an objective cortical marker to delineate prognostic variability between ALS motor phenotypes and help clarify clinical outcomes for patients.

## 5. Discussion

The striking clinical variability between ALS motor phenotypes is built on a common chassis of anterior brain motor neurodegeneration unified by common cellular and molecular pathology. It has been argued that clinical phenotypic discriminators may become increasingly anachronistic in this context, but their variable impact on disease trajectory make these classifications useful stratifiers for predicting clinical outcomes and understanding mechanisms of cell death. Recognition of the UMN lesion across ALS phenotypes has been challenging to detect in vivo, but cortical excitability is shown to be of critical importance to the pathophysiology of disease across all ALS phenotypes. Unarguably, pathobiology extends beyond these considerations alone, and ALS is etiologically a complex syndrome, but these cortical motor features suggest that variable disease kinetics are at least partly based on cortical onset site, degree of hyperexcitability, and distribution between UMN and LMN levels, challenging the traditional diagnostic and prognostic rubric of ALS that does not account for the cortical excitability profile. Interactions between genetics and the environmental may also contribute to the underlying variability of motor neuron degeneration across the motor phenotypes [22], and the increasingly sophisticated repertoire of whole genome studies and molecular biology may help identify further upstream factors that may regulate hyperexcitability and phenotypic variability. Identifying the earliest changes of disease and prospectively tracking motor pathology in vivo will lend an essential brain map aimed at translating this pathophysiology of cortical spread in a dynamic clinical context, with classical and atypical clinical presentations providing a unique window into neuroanatomic onset and progression.

In developing rationally designed therapies for ALS, the recognised clinical motor pattesrns provide important guidance for future development of this current intransigent problem. The heterogeneity of underlying cortical involvement implies the need for their separate consideration, particularly when considering biomarkers and therapeutic response. Stringent phenotypic classifications may facilitate the identification and evaluation of novel treatment targets for phenotypes that share common cortical patterns. This level of granularity has not been adopted in prior clinical trials and may reveal therapies that are preferentially geared towards subtypes of the disease. With the use of large normative databases, it may be possible to quantify the UMN lesion more sensitivity for each distinct phenotype and integrate this at a single patient level algorithm. This will broaden understanding of the association between natural history trajectories and underlying pathophysiological mechanisms, to complement neuropathological and neurophysiological hypotheses supporting a brain-centered disease onset. As precision medicine matures, being cognizant of clinical phenotypic differences will become ever more relevant for therapeutic endeavours that aim to target regional cortical neurons before cell death propagates.

## Figures and Tables

**Table 1:** Motor cortical function measured using TMS across the phenotypes.

Cortical Parameters Using TMS	Typical Phenotype	Atypical Phenotype			
	Classical ALS	PLS	Flail Leg	Flail Arm	IBP
**Single Pulse**					
RMT (%)	N or ↓; inexcitable (10–20%)	↑↑ or inexcitable (71%)	N	N or ↓	N
CSP (ms)	N or ↓	↓	N or ↓ *	↓	N
CMCT (ms)	N or ↑	N →↑↑	↑	N or ↑	↑
**Paired-Pulse**					
Averaged SICI, 1–7 ms (%)	↓ or ↓↓	↓	N or ↓ *	↓	N **
ICF, 10–30 ms (%)	N or ↑	↑	N	↑	N

N, normal (i.e., comparable to healthy controls). * Abnormal only in the presence of clinical UMN signs; ** Normal when clinically restricted to the bulbar region. Findings are in comparison to healthy controls at first clinical visit (recording from the abductor pollicis brevis).

## Supplementary Materials

The following are available online at https://www.mdpi.com/article/10.3390/brainsci11060715/s1, Table S1: Clinical and demographic features of the motor phenotypes.

## References

[1] Ravits J.M., La Spada A.R. (2009). ALS motor phenotype heterogeneity, focality, and spread: Deconstructing motor neuron degeneration. Neurology.

[2] Dharmadasa T., Henderson R.D., Talman P.S., Al Macdonell R., Mathers S., Schultz D.W., Needham M., Zoing M., Vucic S., Kiernan M.C. (2017). Motor neurone disease: Progress and challenges. Med. J. Aust..

[3] Swinnen B., Robberecht W. (2014). The phenotypic variability of amyotrophic lateral sclerosis. Nat. Rev. Neurol..

[4] Chiò A., Calvo A., Moglia C., Mazzini L., Mora G., PARALS study group (2011). Phenotypic heterogeneity of amyotrophic lateral sclerosis: A population based study. J. Neurol. Neurosurg. Psychiatry.

[5] Eisen A., Braak H., Del Tredici K., Lemon R., Ludolph A.C., Kiernan M.C. (2017). Cortical influences drive amyotrophic lateral sclerosis. J. Neurol. Neurosurg. Psychiatry.

[6] Lemon F., Grifithis J. (2005). Comparing the function of the corticospinal system in different species: Organisational differences for motor specialisation?. Muscle Nerve.

[7] Henderson R.D., Garton F.C., Kiernan M.C., Turner M.R., Eisen A. (2018). Human cerebral evolution and the clinical syndrome of amyotrophic lateral sclerosis. J. Neurol. Neurosurg. Psychiatry.

[8] Ravits J., Paul P., Jorg C. (2007). Focality of upper and lower motor neuron degeneration at the clinical onset of ALS. Neurology.

[9] Huynh W., Simon N.G., Grosskreutz J., Turner M.R., Vucic S., Kiernan M.C. (2016). Assessment of the upper motor neuron in amyotrophic lateral sclerosis. Clin. Neurophysiol..

[10] Swash M., Burke D., Turner M.R., Grosskreutz J., Leigh P.N., Decarvalho M., Kiernan M.C. (2020). Occasional essay: Upper motor neuron syndrome in amyotrophic lateral sclerosis. J. Neurol. Neurosurg. Psychiatry.

[11] Kaufmann P., Pullman S.L., Shungu D.C., Chan S., Hays A.P., Del Bene M.L., Dover M.A., Vukic M., Rowland L.P., Mitsumoto H. (2004). Objective tests for upper motor neuron involvement in amyotrophic lateral sclerosis (ALS). Neurology.

[12] Vucic S., Ziemann U., Eisen A., Hallett M., Kiernan M.C. (2012). Transcranial magnetic stimulation and amyotrophic lateral sclerosis: Pathophysiological insights. J. Neurol. Neurosurg. Psychiatry.

[13] Dharmadasa T., Matamala J.M., Howells J., Vucic S., Kiernan M.C. (2020). Early focality and spread of cortical dysfunction in amyotrophic lateral sclerosis: A regional study across the motor cortices. Clin. Neurophysiol..

[14] Menon P., Geevasinga N., Bos M.V.D., Yiannikas C., Kiernan M.C., Vucic S. (2017). Cortical hyperexcitability and disease spread in amyotrophic lateral sclerosis. Eur. J. Neurol..

[15] Groppa S., Oliviero A., Eisen A., Quartarone L., Cohen V., Mall A., Kaelin-Lang T., Mima S., Rossi G., Thickbroom P. (2012). A practical guide to diagnostic transcranial magnetic sitmulation: Report of an I.F.C.N. Committee. Clin. Neurophysiol..

[16] Zanette G., Tamburin S., Manganotti P., Refatti N., Forgione A., Rizzuto N. (2002). Different mechanisms contribute to motor cortex hyperexcitability in amyotrophic lateral sclerosis. Clin. Neurophysiol..

[17] Ziemann U., Winter M., Reimers C.D., Reimers K., Tergau F., Paulus W. (1997). Impaired motor cortex inhibition in patients with amyotrophic lateral sclerosis. Evidence from paired transcranial magnetic stimulation. Neurology.

[18] Dharmadasa T., Howells J., Matamala J.M., Simon N.G., Burke D., Vucic S., Kiernan M.C. (2021). Cortical inexcitability defines an adverse clinical profile in amyotrophic lateral sclerosis. Eur. J. Neurol..

[19] Shibuya K., Park S.B., Geevasinga N., Menon P., Howells J., Simon N.G., Huynh W., Noto Y.-I., Götz J., Kril J.J. (2016). Motor cortical function determines prognosis in sporadic ALS. Neurology.

[20] Talman P., Duong T., Vucic S., Mathers S., Venkatesh S., Henderson R., Rowe D., Schultz D., Edis R., Needham M. (2016). Identification and outcomes of clinical phenotypes in amyotrophic lateral sclerosis/motor neuron disease: Australian National Motor Neuron Disease observational cohort. BMJ Open.

[21] Kiernan M., Vucic S., Cheah B., Turner M., Eisen A., Hardiman O., Burrell J., Zoing M. (2011). Amyotrophic lateral sclerosis. Lancet.

[22] Chiò A., Moglia C., Canosa A., Manera U., D’Ovidio F., Vasta R., Grassano M., Brunetti M., Barberis M., Corrado L. (2020). ALS phenotype is influenced by age, sex, and genetics. Neurology.

[23] Turner M.R., Scaber J., Goodfellow J.A., Lord M.E., Marsden R., Talbot K. (2010). The diagnostic pathway and prognosis in bulbar-onset amyotrophic lateral sclerosis. J. Neurol. Sci..

[24] Turner M.R., Barohn R.J., Corcia P., Fink J.K., Harms M.B., Kiernan M.C., Ravits J., Silani V., Simmons Z., Statland J. (2020). Primary lateral sclerosis: Consensus diagnostic criteria. J. Neurol. Neurosurg. Psychiatry.

[25] Mitsumoto H., Nagy P., Gennings C., Murphy J., Andrews H., Goetz R., Floeter M., Hupf J., Singleton J., Barohn R. (2015). Phenotypic and molecular analysis of primary lateral sclerosis. Neurol. Genet..

[26] Dharmadasa T., Huynh W., Tsugawa J., Shimatani Y., Ma Y., Kiernan M.C. (2018). Implications of structural and functional brain changes in amyotrophic lateral sclerosis. Expert Rev. Neurother..

[27] Claassen D., Josephs K., Peller P. (2010). The stripe of primary lateral sclerosis: Focal primary motor cortex hypometabolism seen on fluorodeoxyglucose F18 positron emission tomography. Arch. Neurol..

[28] Rowland L.P. (1999). Primary lateral sclerosis: Disease, syndrome, both or neither?. J. Neurol. Sci..

[29] Le Forestier N., Maisonobe T., Piquard A., Rivaud S., Crevier-Buchman L., Salachas F., Pradat P.-F., Lacomblez L., Meininger V. (2001). Does primary lateral sclerosis exist? A study of 20 patients and a review of the literature. Brain.

[30] Gordon P.H., Cheng B., Katz I.B., Pinto M., Hays A.P., Mitsumoto H., Rowland L.P. (2006). The natural history of primary lateral sclerosis. Neurology.

[31] Mills C. (1906). Unilateral ascending paralysis and unilateral descending paralysis: Their clinical varieties and their pathological causes. J. Am. Med. Assoc..

[32] Aran F. (1850). Recherches sur une maladie non encore decrite du systeme musculaire (atrophie musculaire progressive). Arch. Gen. Med..

[33] Visser J., Berg-Vos R.M.V.D., Franssen H., Berg L.H.V.D., Wokke J.H., De Jong J.M.V., Holman R., De Haan R.J., De Visser M. (2007). Disease Course and Prognostic Factors of Progressive Muscular Atrophy. Arch. Neurol..

[34] Wijesekera L.C., Mathers S., Talman P., Galtrey C., Parkinson M.H., Ganesalingam J., Willey E., Ampong M.A., Ellis C.M., Shaw C. (2009). Natural history and clinical features of the flail arm and flail leg ALS variants. Neurology.

[35] Hübers A., Hildebrandt V., Petri S., Kollewe K., Hermann A., Storch A., Hanisch F., Zierz S., Rosenbohm A., Ludolph A.C. (2015). Clinical features and differential diagnosis of flail arm syndrome. J. Neurol..

[36] Burrell J.R., Vucic S., Kiernan M.C. (2011). Isolated bulbar phenotype of amyotrophic lateral sclerosis. Amyotroph. Lateral Scler..

[37] Karam C., Scelsa S.N., MacGowan D.J.L. (2010). The clinical course of progressive bulbar palsy. Amyotroph. Lateral Scler..

[38] Turner M.R., Kiernan M.C. (2012). Does interneuronal dysfunction contribute to neurodegeneration in amyotrophic lateral sclerosis?. Amyotroph. Lateral Scler..

[39] Vucic S., Bos M.V.D., Menon P., Howells J., Dharmadasa T., Kiernan M.C. (2018). Utility of threshold tracking transcranial magnetic stimulation in ALS. Clin. Neurophysiol. Pract..

[40] Vucic S., Nicholson G.A., Kiernan M.C. (2008). Cortical hyperexcitability may precede the onset of familial amyotrophic lateral sclerosis. Brain.

[41] Kujirai T., Caramia M.D., Rothwell J.C., Day B.L., Thompson P.D., Ferbert A., Wroe S., Asselman P., Marsden C.D. (1993). Corticocortical inhibition in human motor cortex. J. Physiol..

[42] Fisher R.J., Nakamura Y., Bestmann S., Rothwell J.C., Bostock H. (2002). Two phases of intracortical inhibition revealed by transcranial magnetic threshold tracking. Exp. Brain Res..

[43] Nihei K., McKee A.C., Kowall N.W. (1993). Patterns of neuronal degeneration in the motor cortex of amyotrophic lateral sclerosis patients. Acta Neuropathol..

[44] Foerster B., Callaghan B., Petrou M., Edden R., Chenevert T., Feldmen E. (2012). Decreased motor cortex y-aminobutyric acid in amyotrophic lateral sclerosis. Neurology.

[45] Mills K.R., Nithi K.A. (1997). Corticomotor threshold is reduced in early sporadic amyotrophic lateral sclerosis. Muscle Nerve.

[46] Cantello R., Gianelli M., Civardi C., Mutani R. (1992). Magnetic brain stimulation: The silent period after the motor evoked potential. Neurology.

[47] Rossini P., Burke D., Chen R., Cohen L., Daskalakis Z., Di Iorio R., Di Lazzaro V., Ferreri F., Fitzgerald P., George M. (2015). Non-invasive electrical and magnetic stimulation of the brain, spinal cord, roots and peripheral nerves: Basic principles and procedures for routine clinical and research application. An updated report from an I.F.C.N. Committee. Clin. Neurophysiol..

[48] Saberi S., Stauffer J.E., Schulte D.J., Ravits J. (2015). Neuropathology of Amyotrophic Lateral Sclerosis and Its Variants. Neurol. Clin..

[49] Vucic S., Kiernan M. (2006). Novel threshold tracking techniques suggest that cortical hyperexcitability is an early feature of motor neuron disease. Brain.

[50] Menon P., Geevasinga N., Yiannikas C., Howells J., Kiernan M.C., Vucic S. (2015). Sensitivity and specificity of threshold tracking transcranial magnetic stimulation for diagnosis of amyotrophic lateral sclerosis: A prospective study. Lancet Neurol..

[51] Menon P., Yiannikas C., Kiernan M.C., Vucic S. (2019). Regional motor cortex dysfunction in amyotrophic lateral sclerosis. Ann. Clin. Transl. Neurol..

[52] Bede P., Bokde A., Elamin M., Byrne S., McLaughlin R., Jordan N., Hampel H., Gallagher L., Lynch C., Fagan A.J. (2012). Grey matter correlates of clinical variables in amyotrophic lateral sclerosis (ALS): A neuroimaging study of ALS motor phenotype heterogeneity and cortical focality. J. Neurol. Neurosurg. Psychiatry.

[53] Schuster C., Kasper E., Machts J., Bittner D., Kaufmann J., Benecke R., Teipel S., Vielhaber S., Prudlo J. (2013). Focal thinning of the motor cortex mirrors clinical features of amyotrophic lateral sclerosis and their phenotypes: A neuroimaging study. J. Neurol..

[54] Ellis C., Simmons A., Andrews C., Dawson J., Williams S., Leigh P. (1998). A proton magnetic resonance spectroscopic study in ALS: Correlation with clinical findings. Neurology.

[55] Ellis C.M., Simmons A., Jones D.K., Bland J., Dawson J.M., Horsfield M.A., Williams S.C.R., Leigh P.N. (1999). Diffusion tensor MRI assesses corticospinal tract damage in ALS. Neurology.

[56] Vucic S., Kiernan M.C. (2007). Abnormalities in cortical and peripheral excitability in flail arm variant amyotrophic lateral sclerosis. J. Neurol. Neurosurg. Psychiatry.

[57] Menon P., Geevasinga N., Yiannikas C., Kiernan M.C., Vucic S. (2016). Cortical contributions to the flail leg syndrome: Pathophysiological insights. Amyotroph. Lateral Scler. Front. Degener..

[58] Weber M., Stewart H., Hirota N., Eisen A. (2002). Corticomotneuronal connections in primary lateral sclerosis (PLS). Amyotroph. Lateral Scler. Other Mot. Neuron Disord..

[59] Kuipers-Upmeijer J., Jager A.E.J.D., Hew J.M., Snoek J.W., Van Weerden T.W. (2001). Primary lateral sclerosis: Clinical, neurophysiological, and magnetic resonance findings. J. Neurol. Neurosurg. Psychiatry.

[60] Geevasinga N., Menon P., Sue C.M., Kumar K.R., Ng K., Yiannikas C., Kiernan M.C., Vucic S. (2015). Cortical excitability changes distinguish the motor neuron disease phenotypes from hereditary spastic paraplegia. Eur. J. Neurol..

[61] Takeda T., Kitagawa K., Arai K. (2019). Phenotypic variability and its pathological basis in amyotrophic lateral sclerosis. Neuropathology.

[62] Turner M.R., Hammers A., Al-Chalabi A., Shaw C., Andersen P.M., Brooks D.J., Leigh P.N. (2007). Cortical involvement in four cases of primary lateral sclerosis using [11C]-flumazenil PET. J. Neurol..

[63] Ince P., Evans J., Knopp M., Forster G., Hamdalla H., Wharton S., Shaw P. (2003). Corticospinal tract degeneration in the progressive muscular atrophy variant of ALS. Neurology.

[64] Mackenzie I.R.A. (2020). Neuropathology of primary lateral sclerosis. Amyotroph. Lateral Scler. Front. Degener..

[65] Ziemann U. (2004). TMS and drugs. Clin. Neurophysiol..

[66] Turner M.R., Hammers A., Al-Chalabi A., Shaw C., Andersen P.M., Brooks D.J., Leigh P.N. (2005). Distinct cerebral lesions in sporadic and ‘D90A’ SOD1 ALS: Studies with [11C]flumazenil PET. Brain.

[67] Cwik V.A., Hanstock C.C., Allen P.S., Martin W.R.W. (1998). Estimation of brainstem neuronal loss in amyotrophic lateral sclerosis with in vivo proton magnetic resonance spectroscopy. Neurology.

[68] Maekawa S., Al-Sarraj S., Kibble M., Landau S., Parnavelas J., Cotter D., Everall I., Leigh P.N. (2004). Cortical selective vulnerability in motor neuron disease: A morphometric study. Brain.

[69] Dharmadasa T., Matamala J.M., Kiernan M.C. (2016). Treatment approaches in motor neurone disease. Curr. Opin. Neurol..

[70] Ravits J., Appel S., Baloh R.H., Barohn R., Brooks B.R., Elman L., Floeter M.K., Henderson C., Lomen-Hoerth C., Macklis J.D. (2013). Deciphering amyotrophic lateral sclerosis: What phenotype, neuropathology and genetics are telling us about pathogenesis. Amyotroph. Lateral Scler. Front. Degener..

[71] Matamala J.M., Geevasinga N., Huynh W., Dharmadasa T., Howells J., Simon N.G., Menon P., Vucic S., Kiernan M.C. (2017). Cortical function and corticomotoneuronal adaptation in monomelic amyotrophy. Clin. Neurophysiol..

[72] Kiernan M.C., Vucic S., Talbot K., McDermott C.J., Hardiman O., Shefner J.M., Al-Chalabi A., Huynh W., Cudkowicz M., Talman P. (2021). Improving clinical trial outcomes in amyotrophic lateral sclerosis. Nat. Rev. Neurol..

[73] Agosta F., Pagani E., Petrolini M., Caputo D., Perini M., Prelle A., Salvi F., Filippi M. (2010). Assessment of White Matter Tract Damage in Patients with Amyotrophic Lateral Sclerosis: A Diffusion Tensor MR Imaging Tractography Study. Am. J. Neuroradiol..

