# Supplementary material for: Cortical Excitability across the ALS Clinical Motor Phenotypes
Source: Brain Sci. 2021 May 28;11(6):715. doi: 10.3390/brainsci11060715 (PMC8230203; doi:10.3390/brainsci11060715)
Supplement: Supplementary file 1 [file brainsci-11-00715-s001.zip › brainsci-1215959-supplementary.pdf]

Supplementary Table 1 Clinical motor phenotypes

Clinical motor phenotype	Clinical features		Clinical site of onset (~% frequency)	Number of all cases (~%)	Age at onset (mean, years)	M:F	Prognosis Median in yrs (10-yr survival rate, %)
	UMN	*LMN*					
**‘Typical’ phenotype:**							
Classical ALS	+	+	UL (34)<br>LL (33)	70-75	56.3-62.8<br>(mean range)	1.65:1<br>1:0.71	2.8 (6.3)
			Bulbar (20)		61.4	0.98:1	2.0 (2.1-3.4)
			Respiratory (1-5)<br>Axial, head drop (rare)		(as above)	6.00:1<br>--	1.4^ (0)<br>--
**‘Atypical’ phenotypes:**							
Primary lateral sclerosis (PLS)	+++	+/-*	LL (81)<br>Bulbar (13)<br>UL (6)	2-5	45-52<br>(mean range)	0.98:1	13.1 (71.1)
Progressive muscular atrophy (PMA)	+/-**	+++	UL (47)<br>LL (30)<br>Shoulder girdle (23)	5	61.1	2.04:1	3.3 (11.5)<br>[Similar to classical forms when generalised (>50% regions)]
Restricted variants							
- Flail arm variant<br>- Flail leg variant	+/-	+++	UL (100)<br>LL (100)<br>[Restricted for ≥12 months]	5-6<br>3-3.5	57.3<br>55.0	4.00:1<br>1.03:1	5.0 (15.8)<br>3.0-5.7 (5.3-12.8)
Isolated bulbar palsy (IBP)	++	+	Bulbar (100)<br>[Restricted for ≥6 months]	1-4	65-68<br>(mean range)	1:3.00	3.4

Supplementary Table 1: Frequency, demographics and survival of the typical and atypical phenotypes. +++, primary feature; +/-, possible but not typical; + or ++, typical to the indicated degree. * No ‘active’ LMN degeneration, but minor denervation may be present on EMG ; ** clinical UMN signs can develop within 18months for PMA. UMN, upper motor neurone; LMN, lower motor neurone; UL, upper limb; LL, lower limb; M, male; F, female. N.B: Reported frequencies differ slightly across populations [1-4].^ mean survival given here.
